# Bioaccumulation and biotransformation of simvastatin in probiotic bacteria: A step towards better understanding of drug-bile acids-microbiome interactions

**DOI:** 10.3389/fphar.2023.1111115

**Published:** 2023-02-09

**Authors:** Maja Đanić, Nebojša Pavlović, Slavica Lazarević, Bojan Stanimirov, Saša Vukmirović, Hani Al-Salami, Armin Mooranian, Momir Mikov

**Affiliations:** ^1^ Department of Pharmacology, Toxicology and Clinical Pharmacology, Faculty of Medicine, University of Novi Sad, Novi Sad, Serbia; ^2^ Department of Pharmacy, Faculty of Medicine, University of Novi Sad, Novi Sad, Serbia; ^3^ Department of Biochemistry, Faculty of Medicine, University of Novi Sad, Novi Sad, Serbia; ^4^ The Biotechnology and Drug Development Research Laboratory, Curtin Medical School and Curtin Health Innovation Research Institute, Curtin University, Bentley, WA, Australia; ^5^ Hearing Therapeutics Department, Ear Science Institute Australia, Queen Elizabeth II Medical Centre, Nedlands, WA, Australia

**Keywords:** pharmacomicrobiomics, gut microflora, simvastatin, bile acids, drug metabolism, drug transport

## Abstract

**Introduction:** Although pharmacogenetics and pharmacogenomics have been at the forefront of research aimed at finding novel personalized therapies, the focus of research has recently extended to the potential of intestinal microbiota to affect drug efficacy. Complex interplay of gut microbiota with bile acids may have significant repercussions on drug pharmacokinetics. However, far too little attention has been paid to the potential implication of gut microbiota and bile acids in simvastatin response which is characterized by large interindividual variations.

**The Aim:** In order to gain more insight into the underlying mechanism and its contribution in assessing the clinical outcome, the aim of our study was to examine simvastatin bioaccumulation and biotransformation in probiotic bacteria and the effect of bile acids on simvastatin bioaccumulation in *in vitro* conditions.

**Materials and methods:** Samples with simvastatin, probiotic bacteria and three different bile acids were incubated at anaerobic conditions at 37°C for 24 h. Extracellular and intracellular medium samples were collected and prepared for the LC-MS analysis at predetermined time points (0 min, 15 min, 1 h, 2 h, 4 h, 6 h, 24 h). The concentrations of simvastatin were analyzed by LC-MS/MS. Potential biotransformation pathways were analyzed using a bioinformatics approach in correlation with experimental assay.

**Results:** During the incubation, simvastatin was transported into bacteria cells leading to a drug bioaccumulation over the time, which was augmented upon addition of bile acids after 24 h. A decrease of total drug level during the incubation indicates that the drug is partly biotransformed by bacterial enzymes. According to the results of bioinformatics analysis, the lactone ring is the most susceptible to metabolic changes and the most likely reactions include ester hydrolysis followed by hydroxylation.

**Conclusion:** Results of our study reveal that bioaccumulation and biotransformation of simvastatin by intestinal bacteria might be the underlying mechanisms of altered simvastatin bioavailability and therapeutic effect. Since this study is based only on selected bacterial strains *in vitro*, further more in-depth research is needed in order to elicit completely the contribution of complex drug-microbiota-bile acids interactions to overall clinical response of simvastatin which could ultimately lead to novel approaches for the personalized lipid-lowering therapy.

## 1 Introduction

Given that each individual has its own unique and relatively stable bacterial composition (“bacterial fingerprint”), gut microbiota has attracted a great deal of attention among the scientific community in the field of personalized medicine. Most research to date has focused on the relationship between the state of the microbiome and disease risk (Cullen et al., 2020). In addition to the role in the pathogenesis of many diseases, pharmacomicrobiomic studies have demonstrated that gut microbiota may also affect the bioavailability, clinical efficacy and toxicity of a wide range of drugs. Therefore, pharmacomicrobiomics has become the focus of many research works as a valuable tool for predicting therapy outcomes (ElRakaiby et al., 2014; Scher et al., 2020; Lazarević et al., 2022).

Intestinal microbiota-drugs interactions are possible at different levels, through both direct and indirect mechanisms, *via* bacterially derived metabolites, modulation of barrier function and regulating the gene expression of different transporters and enzymes in gastrointestinal tract (Wilson and Nicholson, 2017). Since the capability of the gut microbiome to metabolize drugs has been recognized for over 80 years (Fuller, 1937), the most studied interaction is the biotransformation of drugs by bacterial enzymes (Enright et al., 2016; Wilson and Nicholson, 2017; Zimmermann et al., 2019). Gut microbiota performs a wide range of metabolic reactions on drugs that may affect the pharmacokinetics of numerous drugs and the final clinical outcome (Wilson and Nicholson, 2017; Đanić and Mikov 2020). Metabolic transformation of drugs by gut microbiota and probiotic bacteria may result in the production of active, inactive or even toxic metabolites contributing to therapeutic effects or even adverse reactions. While the liver is primarily responsible for the metabolism *via* oxidation and conjugation producing polar and high molecular weight metabolites, the intestinal bacteria is mainly involved in reductive and hydrolytic reactions but deamination, dehydroxylation, decarboxylation, demethylation, deconjugation and proteolysis are also described, therefore representing an extremely important site of first-pass metabolism (Stojančević et al., 2014; Wilson and Nicholson, 2017; Đanić and Mikov, 2020). To date, over one hundred drug molecules have been reported to be metabolized by intestinal bacteria (Zimmermann et al., 2019). However, this number is likely much higher since no large-scale systematic screenings for bacterial metabolism have taken place (Bisanz et al., 2018).

Beyond the increasingly recognized scenario of biotransformation by the bacterial enzymes, in a previously published study, we have demonstrated that drugs may also accumulate in gut bacteria (Đanić et al., 2019). To the best of our knowledge, no previous research had considered drug-bacteria interactions in terms of bioaccumulation so this type of interaction is a completely new aspect that needs special attention in future studies to gain a more in-depth understanding of its influence on drug bioavailability. In a recently published study in the journal Nature, a group of authors has studied the interactions of 15 diverse drugs with 25 common strains of gut bacteria, revealing 70 bacteria-drug interactions that included even 29 entirely novel mechanisms. It was surprising that the majority of the new interactions were the drugs accumulating within the bacteria with or without chemical transformation highlighting the role of the microbiome in drug delivery, effectiveness and safety (Klünemann et al., 2021).

Drugs that are particularly susceptible to the effect of gut microbiota are those characterized by low solubility and/or permeability, or modified-release, thereby contributing to longer residence times of a drug within the gastrointestinal tract wherein biotransformation by intestinal bacteria may occur (Stojančević et al., 2014; Enright et al., 2016). Additionally, functional group analysis suggests that particular chemical structures (such as lactones, nitro, azo and urea groups) predispose drugs for microbial metabolism (Zimmermann et al., 2019). One such candidate is simvastatin, a lipid-lowering drug from a group of statins, which acts as a 3-hydroxy-3-methyl-glutaryl-CoA (HMG-CoA) reductase inhibitor. The simvastatin molecule consists of aromatic backbone attached to dimethylbutanoic acid arm with an ester bond and the ethyl pyranyl arm with a covalent C–C-bond forming a lactone structure which represents an inactive form of the drug (Aura et al., 2011). This, simvastatin is administered as a prodrug while the active carboxylate form of the drug is *β*-hydroxy acid which is produced upon hydrolysis by esterases, paraoxonases and by non-enzymatic hydrolysis in the human body (Geboers et al., 2016). The binding of statins to the catalytic domain of HMG-CoA-reductase is stereoselective and simvastatin *in vivo* rapidly converts to the active (3R, 5R)-3,5-dihydroxypentanoic acids, which in turn inhibit this enzyme (Ye and Devasthale, 2016). It is known that simvastatin undergoes extensive hepatic metabolism *via* various pathways including acid/lactone interconversion. According to the biopharmaceutical classification system (BCS), simvastatin belongs to the second class of drugs characterized by low water solubility and high permeability. For this group of drugs, dissolution rate is a limiting factor for bioavailability (Đanić et al., 2016b). The bioavailability of simvastatin is rather low (less than 5%) due to variable absorption (60%–80%), intensive presystemic elimination and drug efflux pathways (De Angelis, 2004; Korani et al., 2019). Despite the confirmed effectiveness of statins therapy in the prophylaxis and management of cardiovascular disease, a number of studies have drawn attention to large inter-individual variability in response, with roughly a third of treated patients achieving the lipid-lowering goals specified in international guidelines indicating a highly unpredictable therapeutic outcome in patients (Postmus et al., 2014; Trompet et al., 2016). Patient-specific factors like genetic predisposition and the drug characteristics such as low solubility and dissolution rate of simvastatin may only partly explain these differences (Pasanen et al., 2006; Ramsey et al., 2014; Đanić et al., 2016b; Lee and Ho 2017; Karaźniewicz-Łada et al., 2018). There are still difficulties with treatment decisions and personalized approaches to simvastatin therapy remain limited (Reiter-Brennan et al., 2020). Therefore, insights into the additional factors that may affect the efficacy of drug are urgently needed to maximize the clinical response. With an attempt to fill this gap, the research group investigated the link between gut microbiota and simvastatin response and demonstrated that antibiotic treatment not only changed the gut microbiota composition but also attenuated the hypolipidemic effect of simvastatin in hyperlipidemic mice (He et al., 2017). Additionally, the metabolomics analysis showed that higher pre-treatment levels of bacterial derived bile acids correlated with low-density lipoprotein cholesterol (LDL-C) lowering in patients who had a good response to simvastatin (Kaddurah-Daouk et al., 2011). These findings indicate that variability might lie in the interactions with intestinal microbiota, which will be the topic of this research.

A growing body of studies over the last few decades has recognized the role of bile acids in the modulation of drug transport through biological membranes, by affecting their solubility or permeability through biological membranes (Ðanić et al., 2018; Đanić et al., 2021; Mooranian et al., 2021; Pavlović et al., 2022). The final outcome of bile acids on drug transport across the biological membrane depends on many factors including type and structure of bile acids, their hydrophobicity and concentration. It should be noted that bile acids increase the solubility and dissolution rate of non-polar drugs primarily at the levels higher than the critical micellar concentration (CMC), while in submicellar concentrations, they mostly influence the drug transport across the biological membranes (Pavlović et al., 2018). Most common drug-bile salt interaction is ion-pairing and the formed complexes may have either higher or lower polarity compared to the drug molecule itself (Ðanić et al., 2018). The influence of bile acids on drug transport may be achieved through the effect on active and passive transport (Stojančević et al., 2013; Đanić et al., 2021; Pavlović et al., 2022). Much of the current literature pays particular attention to the effect of bile acids on the transport into eukaryotic cells through biological barriers such as the blood brain barrier, skin, buccal, nasal, pulmonary and intestinal membranes (Moghimipour et al., 2015).

Due to the physiological presence of bile acids in the gastrointestinal tract and the complex crosstalk between bile acids and gut microbiota in terms of bacterial biotransformation of bile acids that affects their composition and signaling pathways (Wahlström et al., 2016; Ðanić et al., 2018), it would be of immense importance to study the effect of bile acids on the drug transport into bacterial cells as well.

It is clear that the behavior of prodrugs such as simvastatin in the gastrointestinal tract, which includes interactions with intestinal bacteria and bile acids, may have significant repercussions on intestinal drug absorption, metabolism and overall pharmacokinetics. *In vitro* testing of these interactions is clearly an important step in assessing the final effect on the clinical outcome in patients. To gain more insight into the mechanisms underlying this process, we investigated: 1) simvastatin bioaccumulation in probiotic bacteria in *in vitro* conditions, 2) the influence of different bile acids on the simvastatin bioaccumulation in probiotic bacteria and 3) the biotransformation of simvastatin by probiotic bacteria using a bioinformatics approach in correlation with experimental assay.

## 2 Materials and methods

### 2.1 Materials and reagents

Commercial capsules of probiotics (PROBIOTIC^®^, Ivančić i sinovi d. o.o, Serbia) containing 5 × 10^9^ lyophilized cells of *Lactobacillus acidophilus* Rosell-52, *Lactobacillus rhamnosus* Rosell-11 and *Bifidobacterium longum* Rosell-175 strains, were used in the study as representatives of intestinal microbiota. The accuracy of label claims was confirmed by pretesting the number of viable bacteria in capsules using traditional methods of cultivation. Bacterial strains have been identified and characterized by Pasteur Institute, France. Simvastatin was obtained from Hemofarm AD, Serbia. Cholic acid (CA) and deoxycholic acid (DCA) were purchased from Sigma Chemicals Co., St Louis, MO, United States while 12-monoketocholic acid (MKC) was synthesized at the Department of Pharmacology, Toxicology and Clinical Pharmacology, Faculty of Medicine, Novi Sad, Serbia according to the previously published method of Miljkovic et al. (Miljkovic et al., 1996). Water, acetonitrile, ethanol and formic acid were of LC-MS grade and obtained from J.T. Baker (Phillipsburg, NJ, United States). Phosphate buffered saline pH 7.4 was purchased from Gibco, Life Technologies, Grand Island, NY, United States.

### 2.2 Preparation of stock standard and working standard solutions

The stock solution of simvastatin (5 mg/ml) was prepared by dissolving the appropriate amount of simvastatin in ethanol. A series of standard solutions was prepared using the appropriate dilution of stock solution in PBS buffer:AcN (1:4) to reach the final concentrations in the range of 0.625–20 μg/ml. These standard solutions were used for the determination of linearity and the construction of the calibration curve. The dependence of the peak area on the concentration was analyzed. The calibration curve equation was y = 2621x+33447. The correlation coefficient of the calibration curve was *R* ^2^ = 0.9974.

Stock solutions of DCA, 12-MKC and CA at concentrations of 25 mM were prepared by dissolving the appropriate amount of respective bile acids in DMSO. In this study, submicellar concentrations of bile acids were used (0.25 mM) (Natalini et al., 2007).

### 2.3 Experimental protocol and sample preparation

5 × 10^9^ probiotic bacteria were mixed and shaken with 10 ml of simvastatin solution in PBS buffer (50 μg/ml) in a test tube with a screw cap making suspension of probiotic bacteria (5 × 10^8^/mL). Experimental groups with probiotics were labeled with SP, SPD, SPM and SPC (without bile acids, with DCA, 12-MKC and CA, respectively). Control groups were prepared in the same way but without probiotic bacteria (S, SD, SM, SC, respectively) in order to distinguish the spontaneous degradation of simvastatin during the time from the effect of probiotics.

The tubes were incubated at anaerobic conditions at 37°C for 24 h, gently shaking the tubes occasionally. Extracellular and intracellular medium samples were collected and prepared for the LC-MS analysis at predetermined time points (0 min, 15 min, 1 h, 2 h, 4 h, 6 h, 24 h) according to previously published procedure (Đanić et al., 2019). In each time point, after the gentle shaking the tubes to uniformly distribute the content, 100 μL of samples were withdrawn and centrifuged for 5 min at 15,000 rpm to precipitate bacteria. Precipitated bacteria were used for the analysis of intracellular content and the remaining supernatant were carefully poured off and used for analysis of extracellular content. Precipitation of the proteins in the remaining supernatant was achieved by acetonitrile which was added to supernatant in ratio 1:4. Then, samples were centrifuged for 10 min at +4°C and at 15,000 rpm. The obtained supernatant was used for the analysis of extracellular concentration of simvastatin. An aliquot of 10 μL was directly injected in LC-MS/MS system. Precipitated bacteria that remained after the first step of centrifugation were used for the analysis of intracellular content. Cells were washed three times gently with PBS and resuspended in 100 μL of deionized water followed by ultrasonic disruption that was achieved by three 2-min consecutive ultrasound exposure with 3-min rest intervals between in an ice bath. Bacterial cell debris were then pelleted by a centrifugation step and the supernatant was diluted 5-fold with acetonitrile and centrifuged for 10 min at +4°C and at 15,000 rpm before loaded onto a column for the analysis of intracellular fraction of simvastatin. During the analysis, 5-fold dilution with acetonitrile was considered. The concentrations of simvastatin were analyzed by LC-MS/MS. Total concentrations were calculated theoretically as a sum of extracellular and intracellular concentrations. In order to avoid misinterpretation of results due to possible loss of drug by washing the cells, we have prepared control samples of all studied groups in the last time point and measured the concentrations of simvastatin after lysis of total content which contained the total amount of drug. All experiments were performed in triplicates protected from direct sunlight to prevent photodegradation of the drug.

### 2.4 LC-MS analysis

Sample analysis was performed with The LCQ Fleet ion trap mass spectrometer from Thermo Fisher Scientific, Germany operated in positive electrospray mode. The mass spectrometer was coupled to an HPLC system (Thermo Fisher Scientific, Germany) consisting of a quaternary gradient Surveyor LC pump Plus (Thermo Finnigan) and a Surveyor Autosampler Plus (Thermo Finnigan). The system was controlled by Xcalibur LC/MS software (Thermo Fisher Scientific Corporation, v 2.0.7, 2007). Quantitative analysis was performed using LC-MS according to a previously published method (Gambhirea et al., 2011) with minor modifications. In brief, the analysis was performed using a reverse-phase column Zorbax Eclipse Plus-C18 (150 mm × 2.1 mm, 5 μm, Agilent Technologies, United States), and a guard column Zorbax extend C18 (12.5 mm × 2.1 mm, 5 μm, Agilent Technologies, United States). The mobile phase for isocratic elution consisted of 0.1% formic acid in water and acetonitrile (30:70% v/v), at a constant flow rate of 300 μL/min. The injection volume was 10 μL, the column temperature was 20°C and the duration of one analysis was 15 min. The retention time for simvastatin was 9.73 min. The operating conditions of the mass spectrometer were as follows: temperature of the heated capillary 350°C, sheat gas flow (nitrogen) 32.00, auxiliary gas flow (helium) 8.0 (in arbitrary units), source voltage 5.5 kV, source current 100 μA, 1 micro-scan with a maximum ion injection time of 100 ms. MS analysis was performed in positive ion mode in the whole mass range of m/z 90-600. The ion of the adduct molecule with sodium [M + Na]^+^ was selected for quantitative analysis.

Identification of simvastatin metabolites i.e. qualitative analysis was conducted using LC-MS/MS according to method described previously (Hirth, 2011) with slight modifications. Gradient elution was performed with the mobile phase consisting of 0.1% formic acid (A) and acetonitrile containing 0.1% formic acid (B) starting at 42% B followed by a linear gradient to 90% B over 40 min, holding at 90% B from 40 to 50 min, back to starting conditions (42% B) over 1.5 min, and allowing the column to re-equilibrate to the starting conditions (42% B) during the last 8.5 min period (51.5–60 min). The flow rate was set to 200 μL/min. Samples were stored at 15°C in an Agilent autosampler throughout the analyses.

Mass spectral data were acquired on LCQ Fleet™ Ion Trap Mass Spectrometer (Termo Fischer Scientific, Germany), equipped with an electrospray ionization (ESI) source operated in positive ionization mode and used Xcalibur software (Thermofisher Scientific Corporation, version 2.0.7, 2007) for system operation and data manipulation. MS instrument parameters were optimized by infusing the SV (10 μg/ml) with a syringe pump into the MS source at a flow rate of 5 μL/min with LC solvent.

Two scan events were prescribed to run in the LCQ mass spectrometry. The first event was a full-scan spectrum to acquire data on the on protonated molecules [M + H]^+^ or adducts with alkali metal [M + Na]^+^ within the scan range from m/z 150 to 600. The second scan event was performed using a Data Dependent Scan on [M + H]^+^ or [M + Na]^+^. In this mode, three most abundant mass peaks from the first scan are selected for fragmentation. One μscan was used for data acquisition, and the maximum injection time was 100 ms. Product ion MS/MS scans were performed at normalized collision energy of 30.0 (expressed in relative units, %), the isolation width 2 m/z, the activation q value 0.250, charge state 2 and the activation time 30 ms. For the dependent scans, dynamic exclusion was enabled with the following settings: repeat count 2, repeat duration 15 s, exclusion list size 500, and exclusion duration 60 s.

All data were processed using Qual Browser, which is a part of the Thermofinnigan Xcalibur software in combination with software used for Deconvolution is Automated Mass Spectrometry Deconvolution and Identification System (AMDIS) developed by NIST (National Institute of Standards and Technology).

### 2.5 Analysis of simvastatin biotransformation pathways by bacterial enzymes: Databases and bioinformatics approaches

Potential simvastatin biotransformation pathways were analyzed also using a bioinformatics approach and appropriate software packages.

Simvastatin metabolism was anticipated using freely available MetaPrint 2D tool (http://www.metaprint2d.ch.cam.ac.uk/metaprint2d/), which predicts metabolism through data-mining and statistical analysis of known metabolic transformations reposted in the literature. The atoms of a xenobiotic at which metabolic transformations are centered are termed its ‘sites of metabolism (SOM)’. By uploading the SMILES string of simvastatin molecule, MetaPrint2D predicted metabolic reactions and sites of a molecule that are most likely to undergo the metabolism, based on their similarity to known sites of metabolism and sites that are known not to be metabolized. The predicted metabolic sites/atoms are represented by a color code, indicating the probability of biotransformation. For each marked site in the structure, Normalized Occurrence Ratio (NOR) value was generated representing the probability of enzymatic reaction on the particular atom. The most probable SOM is shown in red and the least probable in gray, with probability values ranging from 0 to 1 [red (0.66–1), orange (0.33–0.66), green (0.33–0.15), white (0.15–0.00), and gray (little/no data)] (Carlsson et al., 2010). Additionally, to predict the structures of potential metabolites, the EAWAG-BBD Pathway Prediction System was used (EAWAG-BBD Pathway Prediction System, 2010).

In order to link suggested metabolic pathways with genes and enzymes in tested bacteria, we have searched for different databases which contain a collection of microbial genomes and metabolic pathways. These predictions are based on data on sequenced genomes, computationally inferred data and existing information from the scientific literature (Karp et al., 2019). Databases-driven analysis of bacterial enzymes is an established technique applied in research on drug–microbiota interactions (Liu et al., 2017; Rezazadeh and Babaeipour, 2020; Ankrah and Barker, 2021). The organism search (*Lactobacillus rhamnosus*, *Lactobacillus acidophillus*, *Bifidobacterium longum*) was performed on verified BRENDA, BioCyc and KEGG platforms in order to check their enzymes. Entering a search term resulted in the list of enzymes that was reviewed for the presence of specific enzymes which may act on simvastatin as a substrate.

### 2.6 Statistics

Obtained data were analyzed using statistical software IBM SPSS Statistics, ver. 21 (Systat Software Inc, San Jose, CA, United States). The analysis concerned triplicate results. All data were expressed as mean ± standard deviation (SD). The statistical significance of the difference between the average values of the parameters was tested using one-factor analysis of variance (ANOVA) with Tuckey’s *post-hoc* test for simultaneous comparison of multiple samples, and ANOVA test of repeated measures with the Sidak test for comparing different time points within the same group. Statistical hypotheses were tested at the level of statistical significance of 5% (*p* < 0.05).

## 3 Results

### 3.1 Bioaccumulation of simvastatin into bacterial cells

**FIGURE 1:**
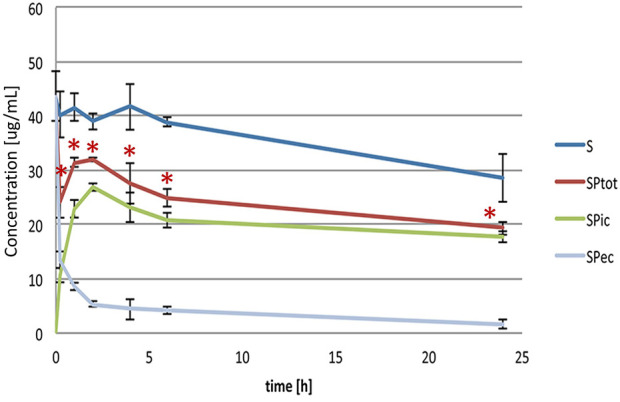
Extracellular, intracellular and total simvastatin level over the 24-h incubation with (SP_ec_, SP_ic_, SP_tot_) and without probiotic bacteria (S). Statistically significant difference of total simvastatin level, calculated as a sum of intracellular and extracellular amount, was noted *versus* control, without probiotic bacteria, during the entire study period (*p < 0.05).

Figure 1 shows the levels of simvastatin in extracellular (SP_ec_), intracellular (SP_ic_) and total (SP_tot_) content during a 24-h incubation with probiotic bacteria compared to the control (S), without probiotic bacteria. It can be observed that during the incubation of simvastatin with probiotic bacteria there was a statistically significant decrease of the simvastatin level in the extracellular content (SP_ec_), which was the most pronounced in the first 15 minutes (from 43.60 ± 4.55 μg/ml at 0 min to 13.38 ± 1.51 μg/ml at 15 min, *p* < 0.05). This trend of decreasing the concentration of simvastatin in the extracellular content continued throughout the entire observed period, but to a lesser extent.

Accordingly, already after 15 minutes of incubation, the presence of simvastatin in the intracellular medium was recorded (10.72 ± 1.37 μg/ml). The concentration continued to increase during the first 2 hours of incubation reaching the value 26.82 ± 0.72 μg/ml, after which it remained relatively stable up to the end of the incubation period with a slight drop from the second hour reaching the value 17.69 ± 1.01 μg/ml after 24 h.

The total concentrations of simvastatin, calculated as a sum of extracellular and intracellular concentrations, were statistically significantly lower in the group with probiotics (SP_tot_) compared to the control group without probiotics (S) during the entire study period. The total concentration of simvastatin in the group with probiotics at 24 h was 19.27 ± 1.16 μg/ml compared to the control where the concentration was 28.53 ± 4.37 μg/ml (*p* < 0.05). The level of simvastatin in the control group was relatively stable during the first 6 h of incubation while it fell significantly at 24 h of incubation, reaching approximately 35% lower values compared to the initial concentration (from 43.60 ± 4.55 μg/ml at 0 min to 28.53 ± 4.37 μg/ml at 24 h, *p* < 0.05).

There were no significant differences in any group between total drug content experimentally and theoretically calculated as a sum of intracellular and extracellular concentration. Therefore, we can conclude that the decrease of a drug is a result of metabolic biotransformation.

### 3.2 Effect of bile acids on the bioaccumulation of simvastatin by bacterial cells

**FIGURE 2:**
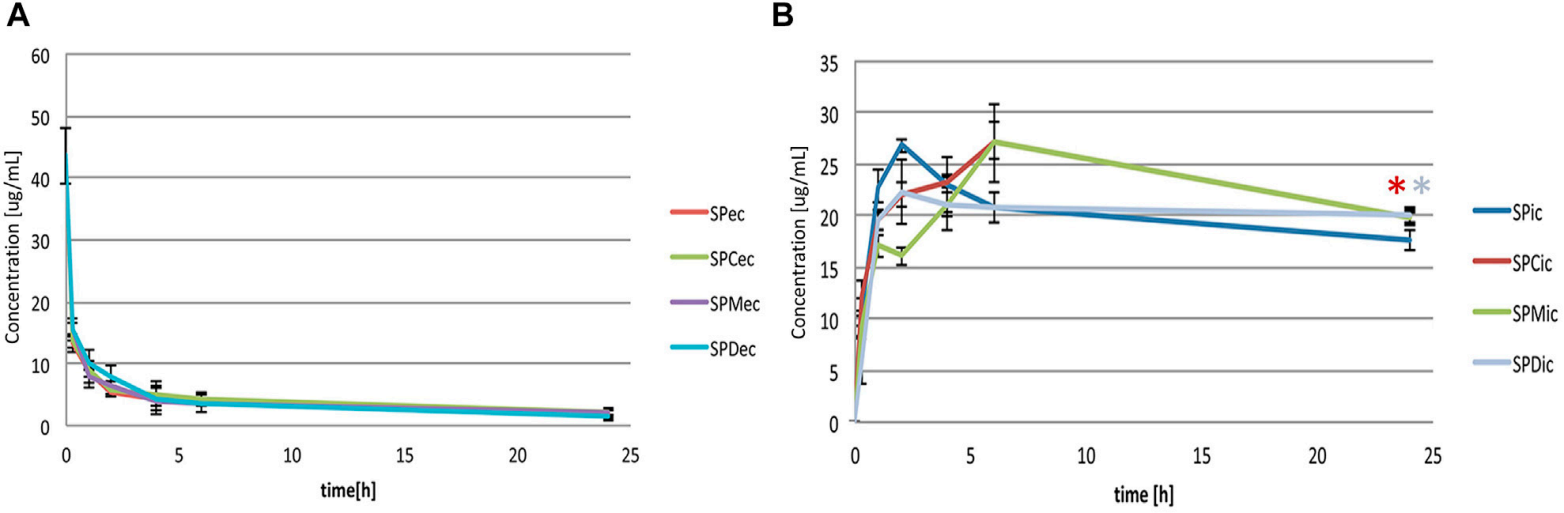
**(A)** Effect of bile acids on extracellular simvastatin concentration over the 24-h incubation (SP_ec_ - simvastatin with probiotics; SPC_ec_, SPM_ec_, SPD_ec_ - simvastatin with probiotics and addition of CA, 12-MKC, DCA, respectively) **(B)** Effect of bile acids on bioaccumulation of simvastatin by probiotic bacteria over the 24-h incubation (SP_ic_ - simvastatin with probiotics; SPC_ic_, SPM_ic_, SPD_ic_ - simvastatin with probiotics and addition of CA, 12-MKC, DCA, respectively). Bile acids did not lead to a significant change in the concentrations of simvastatin in extracellular medium during the 24-h incubation. During the first 4 hours of incubation with probiotic bacteria, statistically lower intracellular concentrations were observed in groups with bile acids compared to the control group. At the end of incubation higher levels of simvastatin in groups with bile acids were recorded (in groups with CA and DCA with statistically significant difference compared to the control, *p < 0.05) with no significant differences between bile acids themselves.

Extracellular and intracellular concentrations of simvastatin in groups with probiotic bacteria and bile acids (SPC, SPM, SPD) were compared to the group without bile acids (SP) and shown in Figures 2A, B, respectively. LC-MS chromatograms for tested groups are provided as Supplementary Data (Supplementary Figure S1). In order to make a comparison between the certain bile acids and to avoid their membranolytic effect, concentrations of bile acids in the experiment were equimolar 0.25 mM and under their CMC (4.09 mM, 13.35 mM and 1.69 mM for CA, 12-MKC and DCA, respectively) (Yang et al., 2009).

It can be observed that bile acids did not generally lead to a significant change in the concentrations of simvastatin in extracellular medium during the 24-h incubation. However, statistically lower intracellular concentrations were observed in groups with bile acids compared to the control group during the first 4 hours of incubation. On the other hand, higher levels of simvastatin in groups with bile acids compared to the control were recorded at the end of incubation, with statistical significance in groups with CA and DCA (19.82 ± 0.44 μg/ml vs. 17.69 ± 1.01 μg/ml and 20.02 ± 0.93 μg/ml vs. 17.69 ± 1.01 μg/ml, *p* < 0.05, respectively) with no significant differences between bile acids themselves.

### 3.3 Biotransformation of simvastatin: Databases and bioinformatics approaches

**FIGURE 3:**
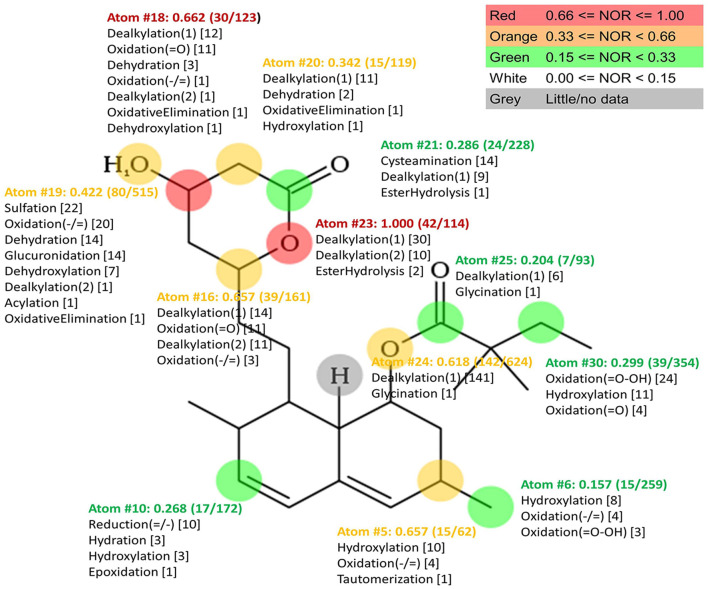
Plot of MetaPrint2D predictions. Atoms which are most likely to be biotransformed and reactions predicted by Meta Print2D are marked. NOR value for each marked atom is assigned indicating the probability of predicted reaction occurring. The most likely metabolic reaction sites are atoms with the highest NOR value which are colored in red.

In order to predict potential microbial metabolic pathways of simvastatin, *in silico* bioinformatics analysis using the MetaPrint2D Program was performed. The results of the analysis are shown in Figure 3. The atoms in the simvastatin molecule that are most susceptible to metabolic reactions are marked, as well as reactions that can take place on them. For each atom marked, NOR value is assigned indicating the probability of predicted reactions occurring. It can be observed that the most likely metabolic reaction sites are atoms with the highest NOR value which are colored in red. In simvastatin molecule, such atoms belong to the lactone ring and the most likely reactions are dealkylation and ester hydrolysis at atom O23, which lead to lactone ring opening, as well as dealkylation, dehydration, dehydroxylation, oxidation and oxidative elimination at C18 atom. Structures of simvastatin metabolites predicted by EAWAG-BBD Pathway Prediction System are shown in Supplementary Figure S2. The presence of enzymes that can catalyze these reactions in tested bacterial strains was confirmed by searching various databases and existing literature.

### 3.4 Biotransformation of simvastatin: Experimental assay

**FIGURE 4:**
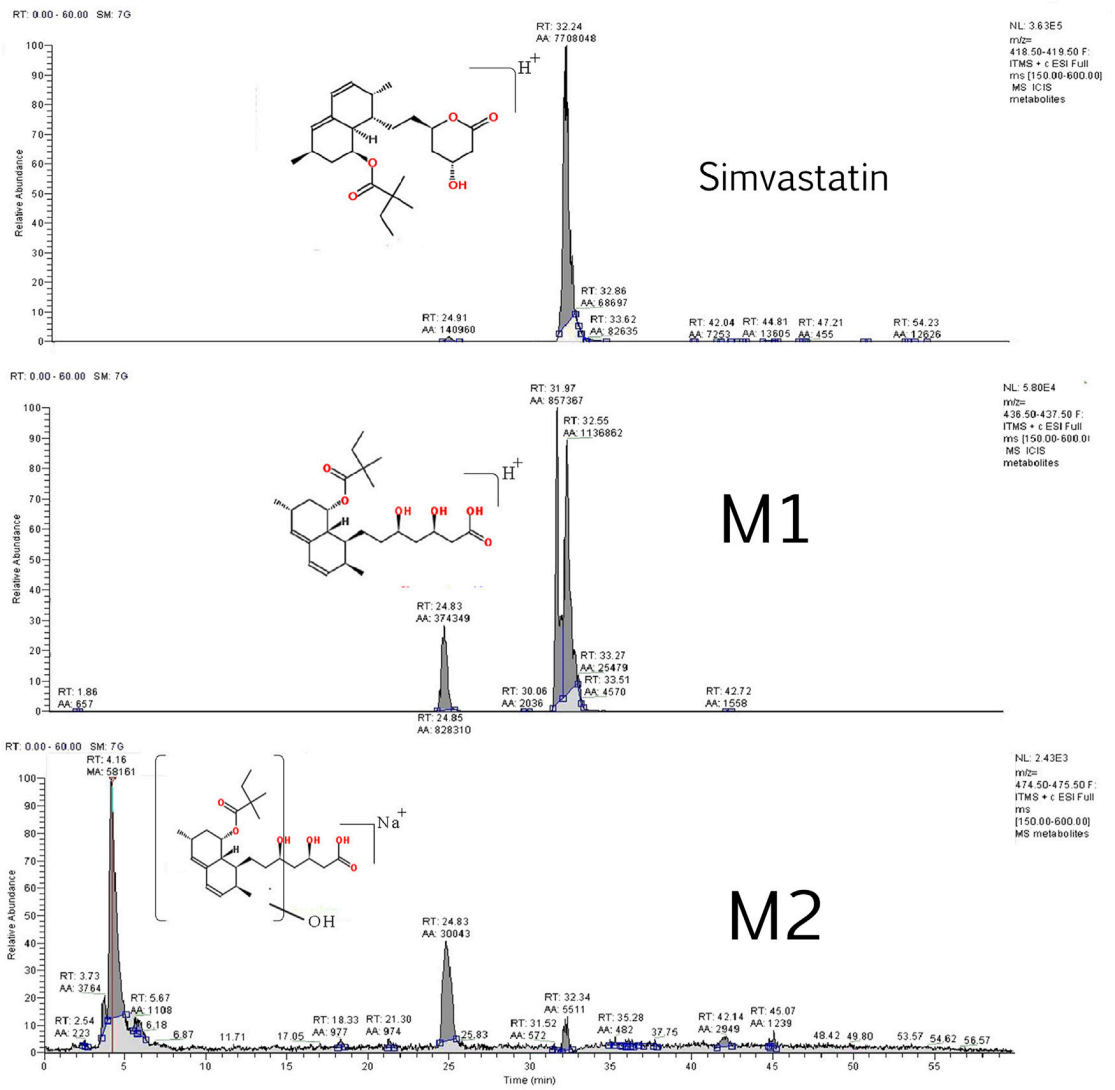
Representative extracted ion chromatograms for simvastatin and its metabolites M1 and M2.

**FIGURE 5:**
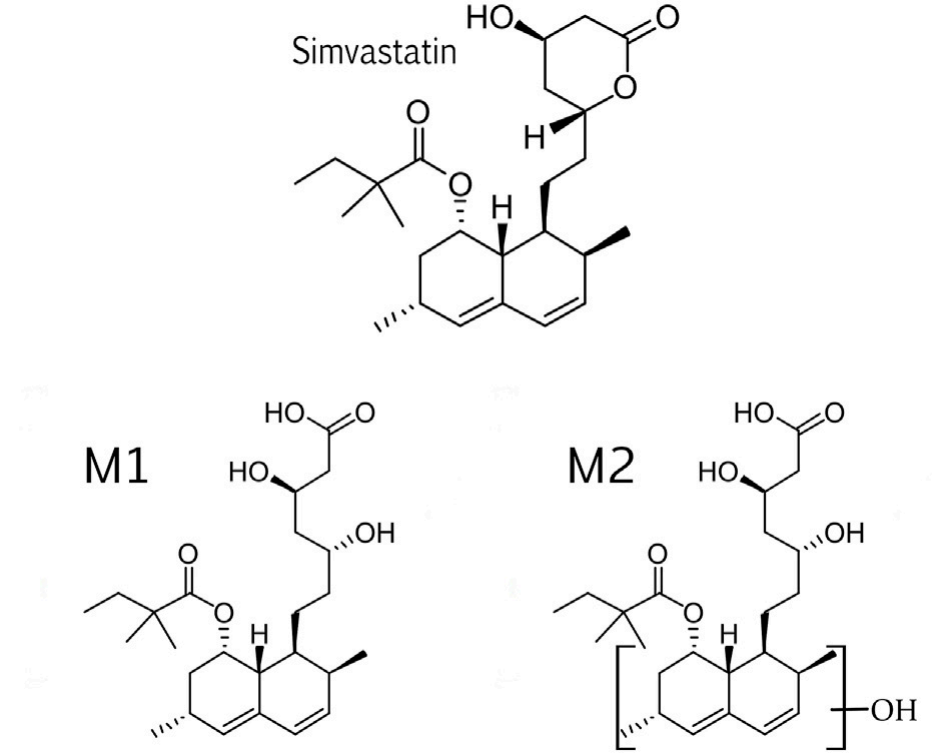
Structures of simvastatin and its metabolites, M1 and M2.

Two simvastatin metabolites were detected in incubation medium. Representative extracted ion chromatograms for simvastatin and its metabolites M1 and M2 are shown in Figure 4 and their structures are represented in Figure 5. The relevant ms1 and MS/MS spectra were provided as Supplementary Figure S3. First metabolite (labeled as M1) was identified as an hydroxy acid metabolite based on a molecular weight increase of 18 Da compared to simvastatin molecule, and its fragmentation pattern. This metabolite represents the open acid form resulted from hydrolysis of lactone. The fragmentation pattern is similar with that of simvastatin with the noticeable fragment ion located at m/z 321 that corresponds to neutral loss of a side chain i.e. 2,2-dimethyl butyric acid molecule (m/z = 116) from molecular ion, at m/z 437. Detected metabolite (labeled as M2) was postulated as a hydroxylated hydroxy acid metabolite, based on a molecular weight increase of 34 Da compared to simvastatin molecule and its fragmentation pattern. Molecular weight of M2 metabolite was determined from the ms1 spectra in the positive mode, through the assignment of molecule adducts with proton [M + H]^+^ (m/z 453) and ions of alkali metals [M + Na]^+^ (m/z 475) and [M + K]^+^ (m/z 491). As the most intense ions from a full MS scan was molecule adduct with Na, it was selected for fragmentation. MS2 spectra shows ion peaks located at 359 and 341 that correspond to the loss of side chain i.e. 2,2-dimethylbutyric acid molecule (m/z 116) and water molecule (m/z 18) from [M + Na]^+^ (m/z 475), respectively. It may be concluded that hydroxylation occurs in naphthalene ring but the accurate position of OH group was not possible to determine. Based on results of MetaPrint 2D analysis, carbon atoms 5, 6 and 10 are the potential targets for hydroxylation. Structures of simvastatin and identified metabolites are shown in Figure 5.

## 4 Discussion

Simvastatin is a drug characterized by large interindividual variations in clinical response and the causes of these differences have not yet been fully elucidated. Although pharmacogenetics and pharmacogenomics have been at the forefront of research examining the variations in drug response in general, the focus of research has been recently extended to the potential of intestinal microbiota to affect drug efficacy. However, far too little attention has been paid to the potential implication of gut microbiota in simvastatin response, and therefore, in the focus of our study were simvastatin-gut microbiota interactions. Considering the lack of information on drug metabolism by gut microbiota generally, the aim of our study was to develop a fast and reliable method that might be useful for pre-evaluation of the gut microbiota impact on drug metabolism and transport using probiotic bacteria from a commercially available product. The first reason is that probiotics are a normal part of gut microbiota and the second one is that they are readily available and do not require special preparation since they contain viable bacteria. Therefore, probiotics are a good option for intestinal microbiota representatives to gain preliminary results on the influence of intestinal bacteria on drug metabolism and pharmacokinetics*.* Potential interactions were studied in *in vitro* conditions during the incubation of simvastatin with selected probiotic strains and using a bioinformatics approach. The influence of bile acids on these interactions was examined too, considering the complex crosstalk between gut bacteria and bile acids and their effects on drugs transport through biological membranes by affecting both, drug solubility and permeability. Both, bacterial biotransformation of simvastatin, and the novel mechanism, drug bioaccumulation, have been reported and discussed.

### 4.1 Bioaccumulation of simvastatin by bacterial cells

Although a grown body of evidence indicate that intestinal microbiota can modulate the availability and efficacy of therapeutic drugs, the systematic mapping of the interactions between drugs and bacteria has only started recently. Much of the research up to now has proposed the biotransformation as the main underlying mechanism of interactions between drugs and bacteria. New aspect that gains a great interest of scientific community is a potential of bacteria to accumulate drugs that may largely affect drug bioavailability and effectiveness (Klünemann et al., 2021). Since there are no previous studies on simvastatin bioaccumulation in intestinal bacteria, in this paper we address this mechanism of potential interaction and its implication in simvastatin action.

From the results obtained which pointed to the increase in intracellular level of drug and decrease in extracellular level, it may be concluded that simvastatin has been transported into bacterial cells leading to a drug bioaccumulation over the time. In addition, a decrease in the total concentration of the drug after 24 h of incubation in the group with probiotic bacteria compared to the control group without bacteria, indicates that the drug has been additionally partly biotransformed by bacterial enzymes (discussed in Section 4.3).

The analysis of the experimental group with probiotic bacteria revealed a significant decrease in the concentration of simvastatin in the extracellular content. At the same time, the drug has been detected in intracellular medium and the concentration rises up to the second hour showing a slight drop after that time. It indicates that the drug has been bioaccumulated by the cells and after some time it has been either metabolized or excreted out from the cells. In addition to the spontaneous release of the drug from the cells, it is assumed that *in vivo* release of the “trapped” drug that remains in the cells will occur only after the cell lysis in the digestive system, which could lead to delayed absorption of simvastatin. Similarly to these results, our previous study demonstrated that antidiabetic drug gliclazide has been transported into probiotic bacteria, being accumulated and partly metabolized through the 24-h incubation (Đanić et al., 2019). Since gliclazide belongs to the same class of drugs as simvastatin according to the BCS, it may be concluded that similar behavior is to be expected from other drugs with low aqueous solubility and good permeability properties. Furthermore, a recently published study has discovered new examples of drugs that accumulate in bacteria including antidepressant duloxetine, anti-diabetic rosiglitazone, antiasthmatic drug montelukast and roflumilast which is used for chronic obstructive pulmonary disease (Klünemann et al., 2021). Some of these drugs were shown exclusively to be accumulated, while the others were found to be both bioaccumulated and metabolized. The different capacity of drugs bioaccumulation by bacterial species is likely due to specificity in uptake and efflux systems between the cells. Given that drug metabolism by bacterial enzymes has long been considered as the major mechanism of how the bacteria affect the fate of drugs in human body, these results have provided potentially additional mechanisms of drug-bacterial interactions that could largely affect drug bioavailability and therapeutic outcome.

### 4.2 The influence of bile acids on simvastatin bioaccumulation by bacterial cells

Given that the final outcome of bile acids on drug transport across the biological membrane depends on many factors including type and structure of bile acids, we selected three of them as representatives; one is representative of hydrophilic bile acids (CA), one is representative of lipophilic (DCA) and the third one is semisynthetic bile acid (12-MKC) that has been synthetized in our laboratory. Although there were no significant differences in simvastatin extracellular levels upon addition of bile acids, there was a lower level of a drug in bacterial cells up to the fourth hour, and higher level at the end of the incubation in groups with bile acids compared to the group without bile acids, thus reaching to the conclusion that bile acids may slow down the bioaccumulation process. It indicates that bile acids could to some extent prevent the delayed absorption of simvastatin that may be caused by “trapping” the drug into bacterial cells. The effect of bile acids on simvastatin transport into the cells may be explained from two different aspects, the effect on active and passive transport. In a previously published study, it was demonstrated that all three studied bile acids led to the decrease of the distribution coefficient of simvastatin in the octanol-buffer system which may be useful in predicting the effect of bile acids on the passive transport of simvastatin across membranes. Decreased distribution coefficient means the reduced affinity for the lipid layer and increased affinity for the aqueous medium, which may explain the reduced drug transport into cells by passive diffusion (Đanić et al., 2016b). Significant insight into the interactions between bile acids and simvastatin has been provided by the computational studies and molecular mechanics calculations where the formation of more hydrophilic aggregates between bile acids and simvastatin has been confirmed, in which bile acids are bonded to simvastatin by hydrophobic interactions, while hydroxyl and keto groups are oriented toward the outer side of the aggregate, thus explaining the higher affinity of the complex for water (Đanić et al., 2016b). Given that the formation of this complex is a reversible process, the free fraction of simvastatin was able to pass through the bacterial membrane, shifting the equilibrium towards the degradation of the complex, thus explaining the slightly higher concentration of simvastatin in bacteria after 24-h incubation. However, the disadvantage of these systems like computational studies and determination of distribution coefficient is the uncertainty of predicting drug behavior in living systems due to additional factors such as the presence of membrane transporters which may be also involved in drug transport across membranes (Kell and Oliver, 2014). Namely, simvastatin may be actively imported into and exported out of the cells *via* bacterial transporters. This assumption is supported by the fact that the same eukaryotic transport proteins (P-gp, MRP2 and OATP1B1) participate in the transport of bile acids and simvastatin (Chen et al., 2005; Klaassen et al., 2010). As there are close homologous proteins of eukaryotic transporters in the bacterial cells with similar substrate specificity (Kourtesi et al., 2013), it is expected that it is possible to achieve competition of simvastatin and bile acids at the level of bacterial transporters as well (Đanić et al., 2016a; Choi et al., 2018). A great affinity of bile acids for a number of so-called multidrug transporters in tested probiotic bacteria has been proved by the molecular docking analyses (Đanić et al., 2016a). Therefore, interactions at the level of transport proteins may be expected. According to our knowledge, this is the very first study examining the uptake of simvastatin into bacterial cells and the effect of bile acids on that process so more in-depth molecular analysis is highly recommended to gain insight into precise transport mechanisms and the affinity of these molecules towards membrane transport proteins in prokaryotic cells.

### 4.3 Analysis of simvastatin biotransformation pathways by bacterial enzymes

A large number of studies have proved that the intestinal microbiota possesses a variety of metabolic activities that are able to modulate the fate of orally administered drugs and their bioavailability. Drug biotransformation by intestinal bacteria may have either a positive or a negative effect on drug activity and efficacy. Although some drugs such as sulfasalazine are converted by microbial enzymes into their active forms, bacterial metabolism can also inactivate drugs such as digoxin, or cause toxic effects as in the case of irinotecan (Stojančević et al., 2014; Swanson, 2015; Sun et al., 2019).

To expand our understanding of simvastatin-microbiota interactions, we sought to determine whether the depletion of a drug in our screen can be explained partly by the microbial biotransformation and gave preliminary insights into these reactions.

In terms of chemical structure, the simvastatin molecule contains an aromatic backbone attached to dimethylbutanoic acid arm with an ester bond and the ethyl pyranyl arm with a covalent C–C-bond forming a lactone structure (Aura et al., 2011). The lactone ring in simvastatin molecule is susceptible to spontaneous hydrolysis, which is pH dependent, being more pronounced in alkaline than in acidic media (Álvarez-Lueje et al., 2005; Malenović et al., 2010; Beltrán et al., 2019). As the experiment was performed at pH 7.4, and the drug level in the control group without probiotic bacteria at the end of the incubation period were 35% lower compared to the initial concentration it is assumed that simvastatin has been partially spontaneously hydrolyzed to the acid. However, during the whole incubation period the total drug level (as a sum of intracellular and extracellular level) in the group with probiotic bacteria was generally significantly lower compared to the control group indicating that in addition to the potential spontaneous degradation, a drug has been also metabolized by the bacterial enzymes. Hydrolysis of the lactone ring and the formation of M1 metabolite i.e. hydroxy acid form of simvastatin may be the also the result of metabolic biotransformation by esterases of probiotic bacteria *Lactobacillus* and *Bifidobacterium* (Arora et al., 1990; Gavini et al., 1991; Brod et al., 2010). Hydroxylated hydroxy acid metabolite, confirmed by experimental assay as M2 metabolite, may be the result of further metabolic biotransformation of M1 metabolite i.e. subsequent hydroxylation that is in agreement with computational predictions. Additionally, these findings are directly in line with results of a previously published *in vivo* study related to gut microbiota-mediated interactions with lovastatin, which differs only in one methyl group in side chain from simvastatin, showing that upon ester hydrolysis and lactone ring opening, subsequent reaction of hydroxylation is likely to occur (Yoo et al., 2014). The enzymes responsible for this reaction, hydroxylases, have been documented in examined bacteria (Brod et al., 2010; Kim and Oh, 2013; Szaleniec et al., 2018).

## 5 Conclusion

In summary, the results of our study suggest that bioaccumulation and biotransformation of simvastatin by intestinal bacteria might be the underlying mechanisms of altered drug availability and therapeutic effect. It has been shown that bile acids affect the bacterial bioaccumulation of a drug affecting both, the active and passive transport that can consequently reflect on absorption rate of a drug. Obtained results and proposed metabolic pathways may be of the vital importance in further elucidation of microbial implication into simvastatin therapeutic outcome. However, the limitation of this study is that it is based only on selected bacterial strains in *vitro* conditions, and further more in-depth research is thus needed in order to reveal how bioaccumulation and biotransformation of simvastatin by intestinal microbiota manifests inside the human body and to elicit completely the contribution of complex drug-microbiota-bile acids interactions to overall clinical response. Therefore, the results of this study provide a strong rationale for further investigations of the effects of drug-gut microbiota-bile acids interactions outside of the usual box of biotransformation. Mapping these interactions would help us better predict clinical outcome in patients providing a good basis for the optimized personalized therapy with the long-term goal of integrating this information into clinical practice.

## Data Availability

The original contributions presented in the study are included in the article/Supplementary Material, further inquiries can be directed to the corresponding author.
