# Supplementary material for: Bioaccumulation and biotransformation of simvastatin in probiotic bacteria: A step towards better understanding of drug-bile acids-microbiome interactions
Source: Front Pharmacol. 2023 Feb 9;14:1111115. doi: 10.3389/fphar.2023.1111115 (PMC9946981; doi:10.3389/fphar.2023.1111115)
Supplement: Supplementary file 2 [file Image3.pdf]

Supplementary Material

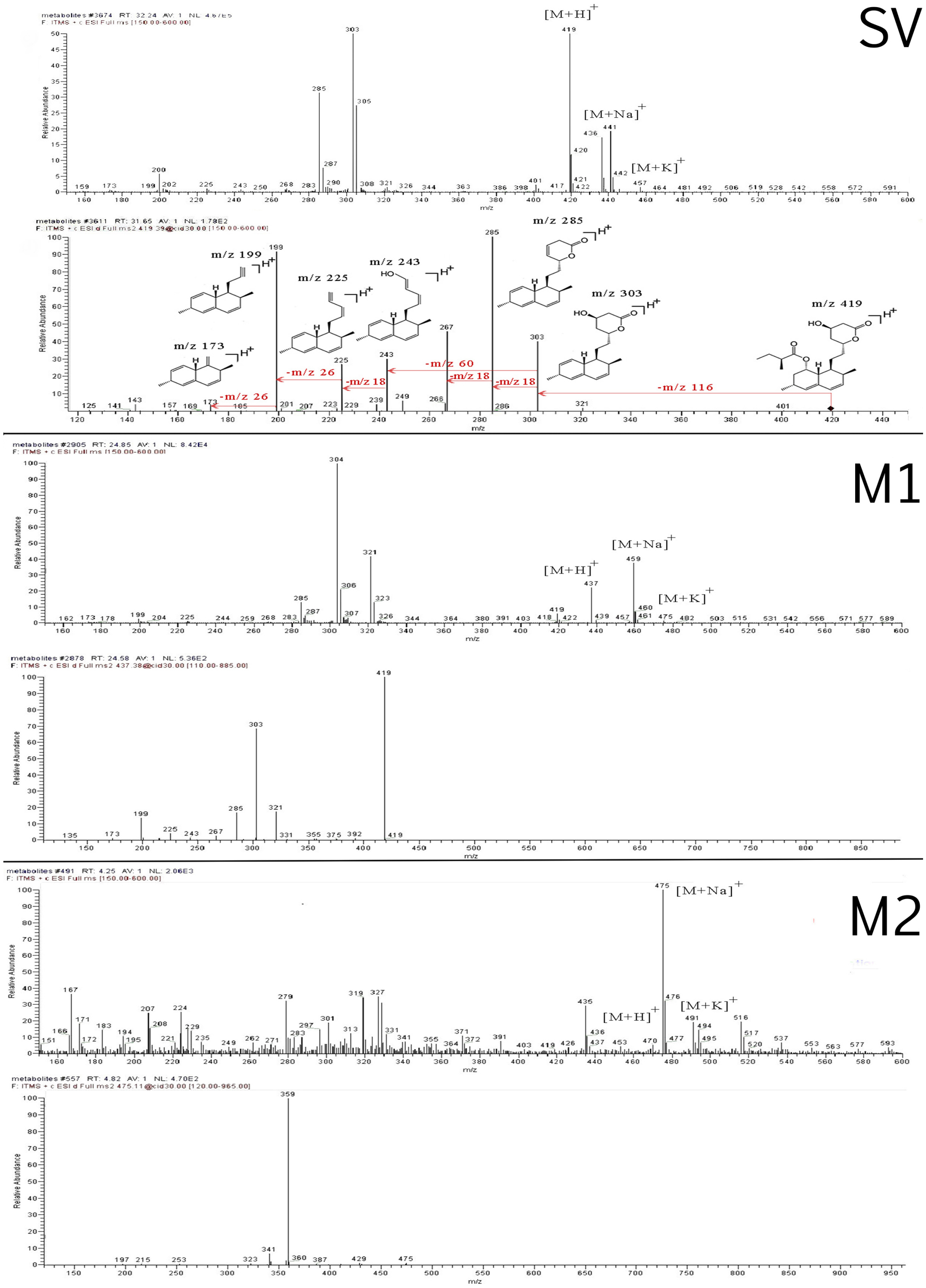

Supplementary Figure 3. The relevant ms1 and MS/MS spectra of simvastatin and its metabolites
