# Supplementary material for: Bioaccumulation and biotransformation of simvastatin in probiotic bacteria: A step towards better understanding of drug-bile acids-microbiome interactions
Source: Front Pharmacol. 2023 Feb 9;14:1111115. doi: 10.3389/fphar.2023.1111115 (PMC9946981; doi:10.3389/fphar.2023.1111115)
Supplement: Supplementary file 3 [file Image1.pdf]

Supplementary Material

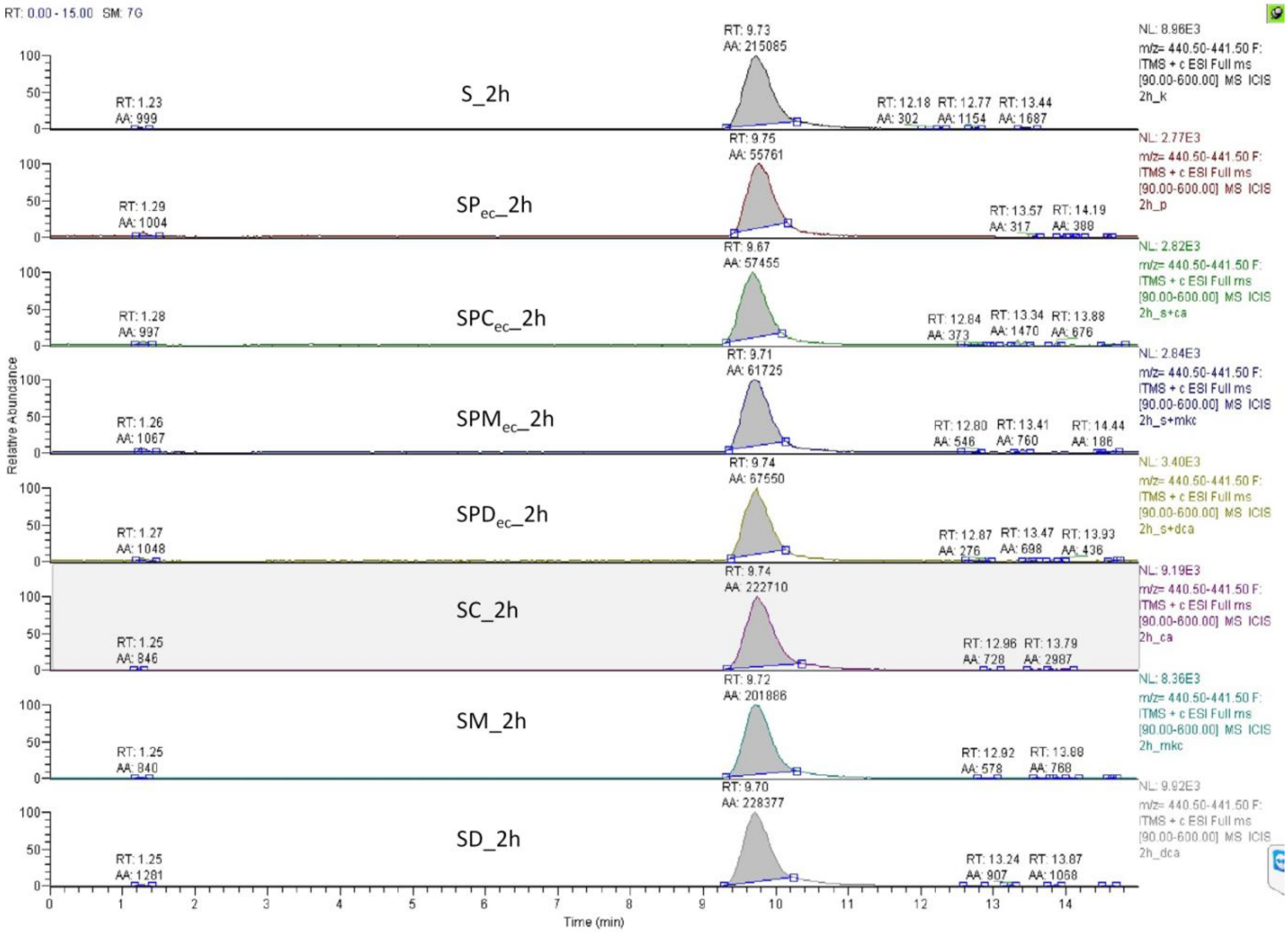

Supplementary Figure 1. LC-MS chromatograms of simvastatin samples after 2h of incubation in extracellular medium with probiotic bacteria and bile acids (S_2h-simvastatin in buffer, SP_{ec}_2h-extracellular concentration with probiotic bacteria, SPC_{ec}_2h- extracellular concentration with probiotic bacteria and CA, SPM_{ec}_2h- extracellular concentration with probiotic bacteria and MKC, SPD_{ec}_2h- extracellular concentracion with probiotic bacteria and DCA, SC_2h- concentration with CA, SM_2h- concentration with MKC, SD_2h- concentration with DCA)
